# Salvianolic Acid A Induces Ferroptosis in Non-Small Cell Lung Cancer via the SRC/YAP/GPX4 Axis

**DOI:** 10.3390/ijms27146265

**Published:** 2026-07-14

**Authors:** Ruyu Jiang, Haoshu Liu, Hairong Xiang, Xiaomeng Tang, Linfeng Zhao, Dawei Zeng, Yue Zhang, Jiazhen Xie, Yanju Gong, Lan Yang

**Affiliations:** 1 College of Basic Medicine, Chengdu University of Traditional Chinese Medicine, Chengdu 611137, China; jiangruyu@stu.cdutcm.edu.cn (R.J.); liuhaoshu@cdzyydx1.wecom.work (H.L.); xianghairong1@stu.cdutcm.edu.cn (H.X.); zhaolinfeng1@stu.cdutcm.edu.cn (L.Z.); zengdawei911@outlook.com (D.Z.); zhangyue2001yy@outlook.com (Y.Z.); jiazhen18796@outlook.com (J.X.); 2 School of Pharmacy, Chengdu University of Traditional Chinese Medicine, Chengdu 611137, China; tangxiaomeng@stu.cdutcm.edu.cn

**Keywords:** Salvianolic acid A, non-small cell lung cancer, ferroptosis, SRC, YAP, GPX4

## Abstract

Lung cancer is the most common malignant tumor worldwide in terms of both incidence and mortality, and the development of highly effective, low-toxicity therapeutic strategies remains an urgent clinical challenge. Here, we report that Salvianolic acid A (SAA), a natural compound extracted from *Salvia miltiorrhiza Bunge*, inhibits the proliferation of non-small cell lung cancer (NSCLC) cells and induces ferroptosis. Mechanistically, SAA acts as an SRC kinase inhibitor, blocking SRC autophosphorylation at Tyr416, thereby disrupting the SRC-YAP interaction and preventing YAP nuclear translocation. This leads to GPX4 downregulation and subsequently triggers ferroptosis, characterized by increased reactive oxygen species (ROS), Fe^2+^ accumulation, and lipid peroxidation. Overexpression of YAP abrogates the effects of SAA, while inhibiting SRC or YAP enhances its activity. SAA inhibits tumor growth and downregulates key effector molecules in vivo. In summary, this study reveals a novel mechanism by which SAA induces ferroptosis via the SRC/YAP/GPX4 axis, supporting its further development as a candidate therapeutic agent for NSCLC.

## 1. Introduction

Lung cancer remains the most frequently diagnosed malignancy and the foremost cause of cancer-associated mortality in China and globally. Non-small cell lung cancer (NSCLC) accounts for approximately 80–85% of all lung cancers. Among patients with advanced disease, the 5-year survival rate remains lower than 5% [1]. In the past two decades, targeted molecular therapy and immunotherapy have greatly improved the prognosis for individuals with NSCLC [2]. However, this disease is still prone to recurrence and metastasis. The main problem is that patients may develop primary or acquired resistance to drugs [3,4,5]. Therefore, discovering new molecular targets and developing effective therapeutic compounds are crucial to improving the treatment of NSCLC.

In the past few years, ferroptosis has been identified as a unique regulatory cell death method. It is characterized primarily by iron-dependent reactive oxygen species (ROS) accumulation, distinguishing it from other cell death pathways such as apoptosis and autophagy [6]. Iron-induced apoptotic cells show typical morphological characteristics, especially partial or complete absence of mitochondrial ridges, damage to the mitochondrial outer membrane, and increased mitochondrial membrane density [7,8]. Dysregulation of ferroptosis is closely related to a variety of human cancers [9,10]. Growing evidence indicates that activating ferroptosis can effectively suppress tumor growth, which highlights its value as an emerging treatment route in the field of oncology [11,12]. Nevertheless, the exact contribution of ferroptosis in NSCLC and its potential therapeutic significance are still not fully understood.

Salvianolic acid A (SAA), derived from the traditional Chinese herb *Salvia miltiorrhiza Bunge*, has exhibited antitumor activity in several different cancer cell types and has been reported to induce apoptotic cell death [13,14,15]. Previous studies have further indicated that SAA is capable of inhibiting angiogenesis by reducing GRP78 secretion, while also limiting metastatic behavior [16,17]. Moreover, SAA enhances chemosensitivity and reverses chemotherapy resistance [18,19]. Nevertheless, the precise antitumor activities of SAA in NSCLC and the molecular mechanisms responsible for its effects remain elusive.

In this research, we explored the treatment impacts of SAA via an integrated strategy that merged network pharmacology analysis with both cellular and animal models of NSCLC. The findings indicate that SAA suppresses the progression of NSCLC by promoting ferroptotic cell death mediated through the SRC/YAP/GPX4 signaling pathway. Overall, the findings support the conclusion that SAA has considerable potential as a novel therapeutic strategy for NSCLC.

## 2. Results

### 2.1. SAA Suppresses the Growth of A549 Cells and Triggers Apoptosis

**Figure 1:**
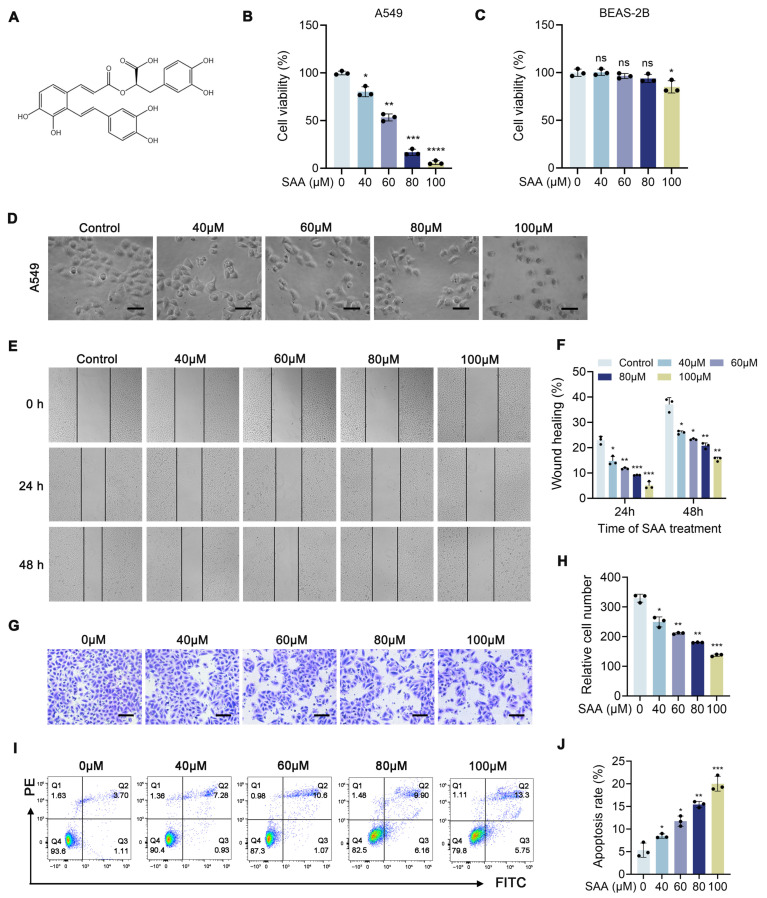
SAA inhibits the growth of A549 cells and triggers apoptosis. (**A**) Chemical structure of SAA. (**B**) CCK-8 assay determined the cytotoxicity of SAA (0, 40, 60, 80 and 100 μM) in A549 cells for 24 h (*n* = 3). (**C**) CCK-8 assay determined the cytotoxicity of SAA in BEAS-2B cells for 24 h (*n* = 3). (**D**) Representative images of SAA in A549 cells for 24 h. Scale bar: 50 μm. (**E**,**F**) Cell migration was assessed using the wound healing assay. (**G**,**H**) Transwell assay. Scale bar: 100 μm. (**I**,**J**) Apoptosis in A549 cells after 24 h treatment with SAA was quantified via flow cytometry (*n* = 3). **** *p* < 0.0001, *** *p* < 0.001, ** *p* < 0.01, * *p* < 0.05 vs. control.

To evaluate the cytotoxic and growth-inhibitory effects of SAA on A549 cells, the human NSCLC cell line A549 was treated with SAA (Figure 1A). First, the cells were incubated in SAA solutions of various concentrations for 24 h, and their viability was then measured using the CCK-8 assay. The findings demonstrated that SAA markedly inhibited the proliferation of A549 cells (Figure 1B). Interestingly, SAA-mediated growth inhibition was not observed in normal human bronchial epithelial BEAS-2B cells (Figure 1C), indicating that SAA exhibits selective cytotoxicity toward A549 cells over BEAS-2B cells. The IC_50_ values were 58.85 μM for A549 cells and 168.6 μM for BEAS-2B cells (Figure S1). Subsequently, cell proliferation was assessed following SAA treatment. Consistently, SAA-mediated growth inhibition of A549 cells was further validated through microscopy images (Figure 1D). Second, we conducted a wound healing assay to evaluate whether SAA affects cell migration. The results showed that SAA could obviously weaken the migration of A549 cells in a time- and dose-dependent manner (Figure 1E,F). Third, to further verify its effect on cell invasion, a Transwell invasion assay was performed following SAA treatment. The relevant results indicated that the number of cells passing through the membrane declined progressively as the SAA concentration increased (Figure 1G,H). Following double staining with Annexin V-FITC and PI, flow cytometric analysis revealed a marked, concentration-dependent rise in both early and late apoptotic A549 cells (Figure 1I,J). Overall, these findings demonstrate that SAA effectively inhibits proliferation, migration, and invasion of A549 cells while promoting apoptosis.

### 2.2. Prediction of the Potential Mechanism of SAA in the Treatment of NSCLC

**Figure 2:**
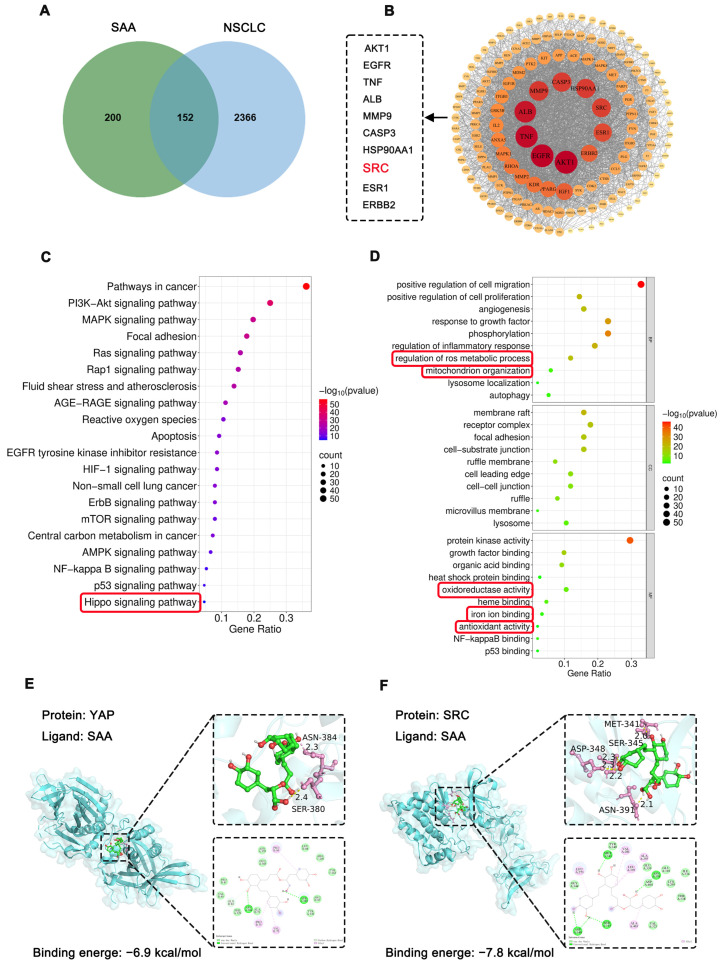
Network pharmacological analysis of SAA in treatment of A549 cells. (**A**) Venn diagram of SAA component target and NSCLC disease target. (**B**) PPI network of intersection targets in NSCLC after SAA treatment. (**C**) Enrichment analysis of potential signaling pathways based on KEGG. (**D**) GO analysis. (**E**) Predicted interaction mode between SAA and YAP. (**F**) Predicted interaction mode between SAA and SRC.

To comprehensively investigate the possible pharmacological mechanisms involved, candidate target proteins were inferred through the SwissTargetPrediction and PharmMapper databases. Meanwhile, genes related to NSCLC were retrieved from the GeneCards, DrugBank, and OMIM databases. After integrating the data and removing duplicates, 152 common targets between SAA and NSCLC were identified (Figure 2A). We constructed a PPI network using the STRING 11.5 database and visualized it in Cytoscape (Figure 2B). The 10 most important nodes were AKT1, EGFR, TNF, ALB, MMP9, CASP3, HSP90AA1, SRC, and ESR1. Subsequently, the 152 common target genes were further subjected to GO and KEGG enrichment evaluation. Enrichment analysis of KEGG pathways identified the 20 most frequent signaling pathways (Figure 2C). Among these, reactive oxygen species (ROS), ErbB, P53, and Hippo were identified as major signaling pathways. GO analysis results revealed significant enrichment in ROS response, oxidative stress, and iron ion-related terms (Figure 2D). These findings indicated that SAA may promote A549 cell death via ferroptosis.

Recent evidence has reported that YAP expression levels are significantly elevated in the tissues and cells of NSCLC [20]. Additionally, a previous study has demonstrated that SAA inhibits the growth in nasopharyngeal carcinoma by inhibiting SRC activation [21]. Importantly, reduction in SRC expression has been shown to alleviate drug resistance in individuals with NSCLC [22,23]. In addition, SRC serves as an upstream regulatory factor in the Hippo signaling pathway and can influence the function of YAP, a major effector located downstream of the pathway [24,25,26]. To confirm whether SAA interacts with SRC and YAP, we conducted molecular docking. The results revealed that SAA forms stable structures with both SRC and YAP, with binding energies of −6.9 kcal/mol for YAP and −7.8 kcal/mol for SRC (Figure 2E,F). SAA formed hydrogen bonds with amino acid residues of YAP, including ASN-384 and SER-380, with bond distances of 2.3 Å and 2.4 Å. In addition, SAA also formed multiple hydrogen bonds with SRC, involving MET-341, ASN-391, SER-345, and ASP-348 with bond distances of 2.0 Å, 2.3 Å, 2.2 Å, and 2.1 Å. These molecular docking results suggested the direct binding of SAA to YAP and SRC. To validate the reliability of the docking, redocking of the co-crystallized ligands into their respective protein structures was performed. Root Mean Square Deviation (RMSD) analysis was 0.644 Å for SRC and 1.987 Å for YAP, confirming the accuracy and reproducibility of the docking parameters (Figure S2). Therefore, we hypothesize that SAA may induce ferroptosis in A549 cells by modulating the SRC/YAP signaling axis.

### 2.3. SAA Promotes Ferroptosis in A549 Cells

**Figure 3:**
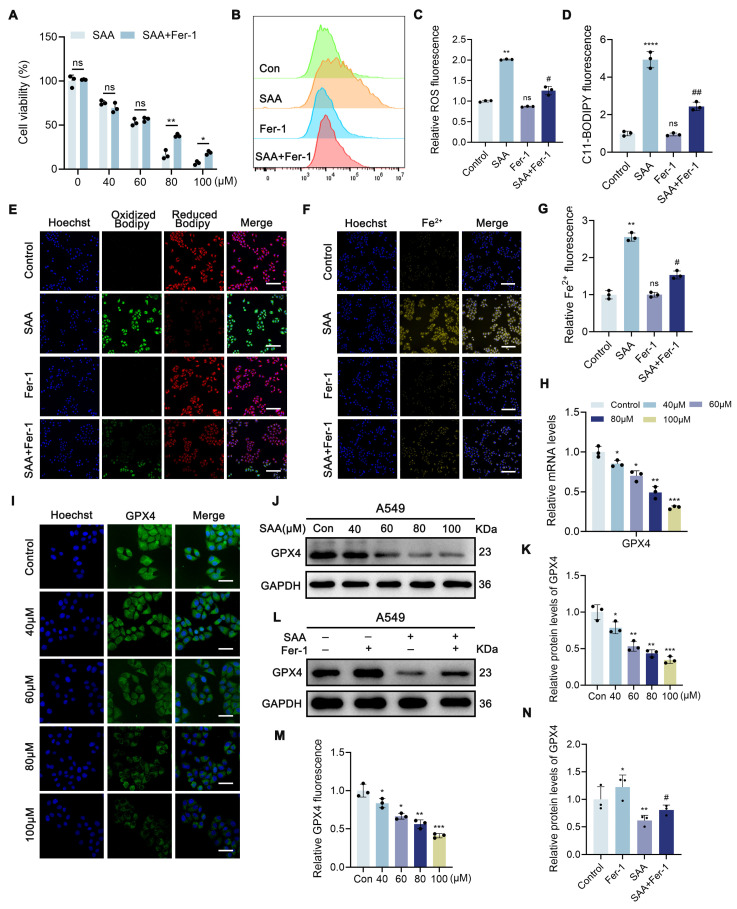
Ferroptosis serves as a critical regulatory factor in SAA-induced cell death in A549 cells. (**A**) CCK-8 assay determined the cytotoxicity of SAA (0, 40, 60, 80 and 100 μM) with or without Fer-1 (5 μM) in A549 cells for 24 h (*n* = 3). (**B**,**C**) Intracellular ROS levels in A549 cells. (**D**,**E**) Lipid peroxidation levels in A549 cells. Scale bar: 100 μm. (**F**,**G**) Intracellular Fe^2+^ levels in A549 cells. Scale bar: 100 μm. (**H**) GPX4 mRNA expression in A549 cells assessed via RT-qPCR. (**I**,**M**) IF staining of GPX4. Scale bar: 25 μm. (**J**,**K**) GPX4 expression in A549 cells following SAA treatment was assessed using Western blot. (**L**,**N**) GPX4 expression in A549 cells was further examined by Western blotting following treatment with SAA (100 μM) with or without Fer-1 (5 μM). **** *p* < 0.0001, *** *p* < 0.001, ** *p* < 0.01, * *p* < 0.05 vs. control and ^##^ *p* < 0.01, ^#^ *p* < 0.05 vs. SAA (100 μM).

To determine whether ferroptosis was the predominant form of cell death in SAA-treated A549 cells, we utilized Ferrostatin-1 (Fer-1, ferroptosis inhibitor) and Z-VAD-FMK (Z-VAD, apoptosis inhibitor). Notably, the results revealed that Fer-1 significantly protected cells from SAA-induced cell death (Figure 3A). In contrast, the apoptosis inhibitor Z-VAD weakly rescued the anti-proliferative effects of SAA (Figure S3). This suggests that the impact of SAA on cell viability may be related to ferroptosis rather than apoptosis. To further substantiate the finding that SAA induces ferroptosis in A549 cells, we investigated several key features of this process, including ROS production, lipid peroxidation, and Fe^2+^ accumulation (Figure 3B–G). Data showed that SAA treatment led to increased ROS levels, elevated lipid peroxidation, and higher intracellular Fe^2+^. Additionally, Fer-1 alleviated SAA-induced ROS, Fe^2+^ accumulation, and lipid peroxidation. Furthermore, in the IF assay, the fluorescence intensity of GPX4 significantly decreased with increasing SAA concentrations (Figure 3I,M). Concurrently, Western blot and RT-qPCR analyses revealed that treatment with SAA markedly downregulated GPX4 expression (Figure 3H,J,K). Conversely, the decrease in GPX4 caused by SAA was partially reversed after treatment with Fer-1 (Figure 3L,N). Collectively, the above results support the conclusion that SAA effectively promotes ferroptosis in A549 cells, representing a central mechanism for its antitumor effects.

### 2.4. SAA Inhibits SRC, Leading to the Inactivation of YAP

**Figure 4:**
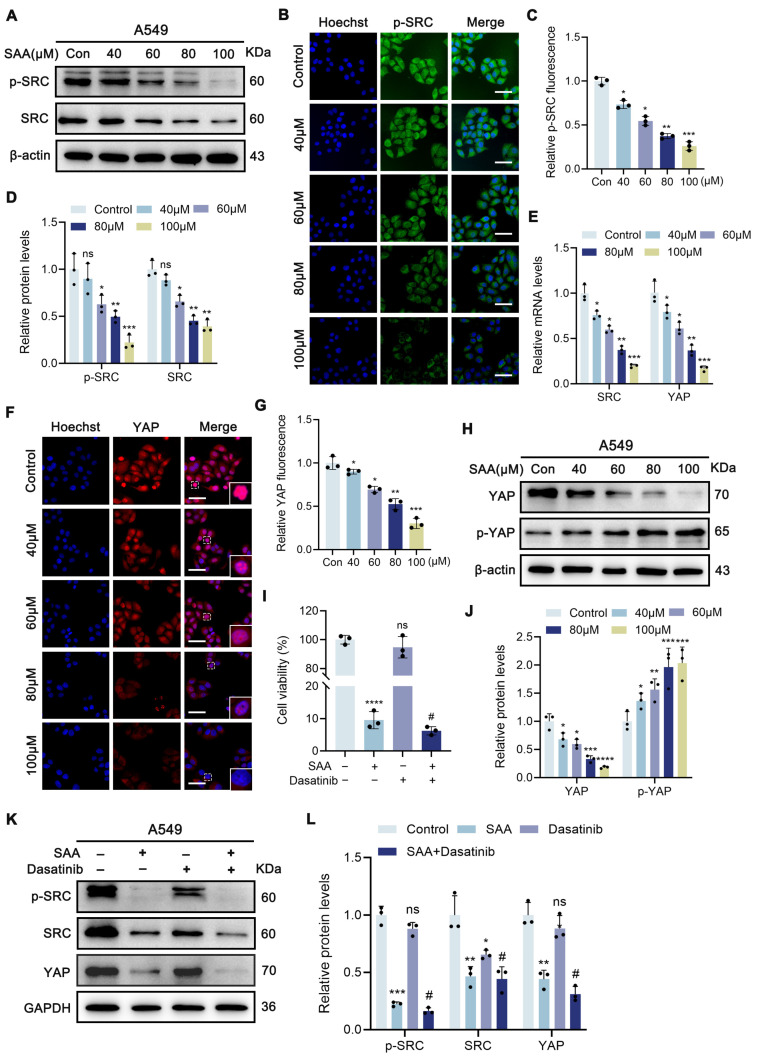
SAA inhibits SRC, leading to the inactivation of YAP. (**A**,**D**) Western blot detection of p-SRC and SRC in A549 cells. (**B**,**C**) IF staining of p-SRC. Scale bar: 25 μm. (**E**) The transcriptional expression of SRC and YAP was quantified in A549 cells via RT-qPCR. (**F**,**G**) IF staining of YAP. Scale bar: 25 μm. (**H**,**J**) Protein expression of YAP and p-YAP was evaluated in A549 cells using Western blot analysis. (**I**) CCK-8 assessing the influence of SAA (100 μM) with or without dasatinib (2 μM) for 24 h on the viability in A549 cells (*n* = 3). (**K**,**L**) Protein expression levels of p-SRC, SRC, and YAP were assessed in A549 cells using Western blot analysis. **** *p* < 0.0001, *** *p* < 0.001, ** *p* < 0.01, * *p* < 0.05 vs. control and ^#^ *p* < 0.05 vs. SAA (100 μM).

Based on network pharmacology analysis and prior evidence implicating SRC and YAP in ferroptosis regulation, we first focused on SRC, an upstream activator of the YAP/TAZ signaling axis. Western blotting showed that SAA significantly reduced p-SRC (Tyr416) and SRC in A549 cells (Figure 4A,D). This inhibition was further corroborated by IF staining, which showed a marked decrease in p-SRC signal within the cytomembrane and cytoplasm (Figure 4B,C). Correspondingly, RT-qPCR confirmed the downregulation of SRC mRNA (Figure 4E). We next examined the downstream effector YAP. Consistent with SRC inhibition, SAA treatment also reduced YAP mRNA levels (Figure 4E). Importantly, IF staining demonstrated that SAA effectively blocked the nuclear translocation of YAP, retaining it predominantly in the cytoplasm (Figure 4F,G). Western blot analysis demonstrated that SAA markedly decreased YAP protein expression in A549 cells, accompanied by a corresponding rise in p-YAP (Ser127) in a dose-dependent manner (Figure 4H,J). Furthermore, to validate whether SRC is required for YAP inactivation, we employed an SRC inhibitor dasatinib. Our findings demonstrated that dasatinib potentiated the SAA-induced reduction in cell viability (Figure 4I). Western blotting revealed that dasatinib effectively increased the inhibitory impact of SAA on p-SRC, SRC and YAP expression (Figure 4K,L). Overall, our results indicate that SAA-mediated YAP inactivation depends on SRC inactivation.

### 2.5. Disruption of the SRC-YAP Interaction by SAA Leads to GPX4 Downregulation and Ferroptosis Induction in A549 Cells

**Figure 5:**
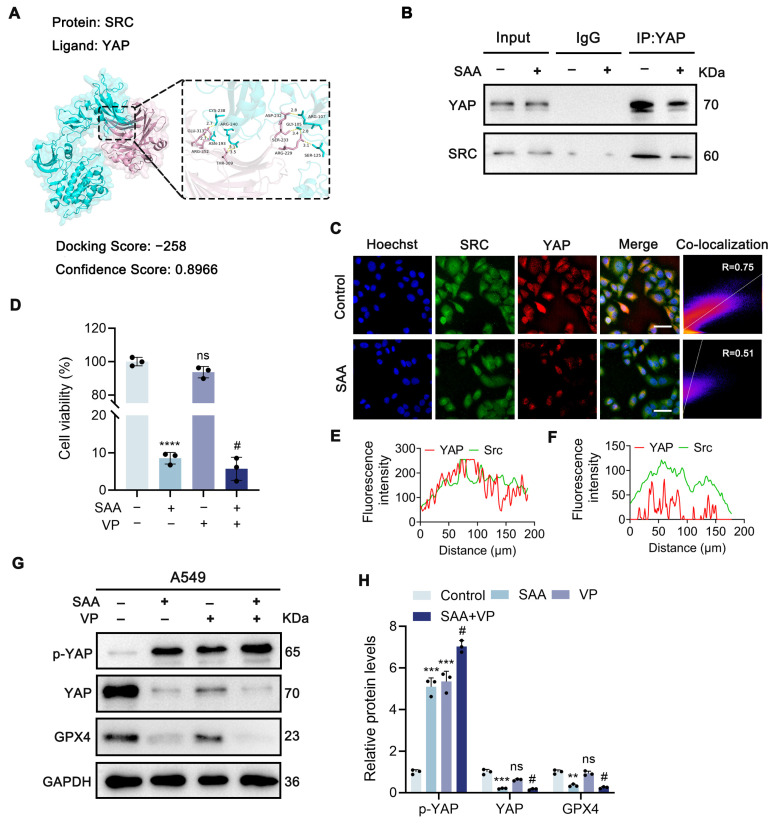
YAP interacts with SRC and modulates ferroptosis in A549 cells. (**A**) Docking analysis of the SRC binding mode to YAP. (**B**) Endogenous Co-IP was performed in A549 cells with anti-YAP antibody, and SRC was subsequently detected. (**C**,**E**,**F**) IF double staining detection of SRC and YAP colocalization in A549 cells. Scale bar: 25 μm. (**D**) To assess cell viability, A549 cells were treated for 24 h with 100 μM SAA either alone or in combination with 2.5 μM VP, followed by the CCK-8 assay. (*n* = 3). (**G**,**H**) The expression of p-YAP, YAP, and GPX4 in A549 cells was analyzed by Western blotting. **** *p* < 0.0001, *** *p* < 0.001, ** *p* < 0.01 vs. control and ^#^ *p* < 0.05 vs. SAA (100 μM).

Studies have shown that SRC not only indirectly activates YAP by phosphorylating LATS1/2, but also directly binds to YAP, thereby enhancing its stability, nuclear localization, and transcriptional activity. Previous emerging evidence has demonstrated that SAA inactivates YAP and downregulates GPX4; we hypothesized that SAA may regulate YAP function by affecting the physical interaction between SRC and YAP. To investigate the potential association between SRC and YAP, molecular docking analysis was conducted, which showed a low binding energy (−258) and high confidence score (0.8966) (Figure 5A). Co-IP assay confirmed a robust endogenous association between SRC and YAP, an interaction which was markedly attenuated by SAA treatment (Figure 5B). Moreover, we assessed their colocalization, and SAA treatment was found to cause a marked separation between the majority of SRC and YAP fluorescent signals (Figure 5C,E,F). Studies have found that YAP1 positively regulates the expression of GPX4 and promotes gastric cancer progression. To further clarify the involvement of YAP in GPX4 regulation in NSCLC, A549 cells were treated with both SAA and the YAP inhibitor verteporfin (VP) (Figure 5D). Our findings demonstrated that VP enhanced the decrease in cell viability caused by SAA. In addition, Western blot analysis indicated that combined treatment with SAA and VP led to a more pronounced decrease in p-YAP, YAP, and GPX4 protein expression compared with SAA treatment alone (Figure 5G,H). These findings support the conclusion that YAP is a key mediator of SAA in NSCLC. These findings suggest that SAA inhibits the SRC-YAP interaction, thereby blocking YAP nuclear translocation, ultimately downregulating GPX4 and triggering ferroptosis.

### 2.6. YAP Overexpression Attenuates SAA-Induced Antitumor Effects and Ferroptosis in A549 Cells

**Figure 6:**
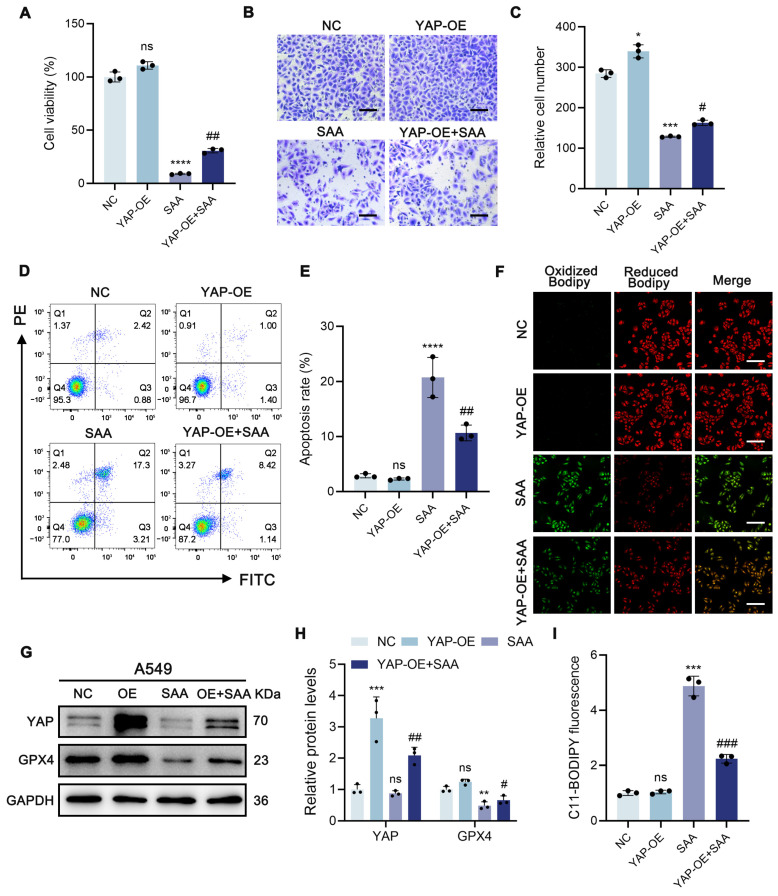
Overexpression of YAP suppresses SAA-induced ferroptosis and attenuates its antitumor effects in A549 cells. (**A**) CCK-8 assay was used to measure the viability of YAP-overexpressing A549 cells, with or without 100 μM SAA treatment. (**B**,**C**) Transwell assay. Scale bar: 100 μm. (**D**,**E**) Following a 24 h exposure to 100 μM SAA, flow cytometry was employed to assess apoptosis in A549 cells with YAP overexpression. (*n* = 3). (**F**,**I**) Measurements of lipid peroxidation were carried out in A549 cells with YAP overexpression. Scale bar: 100 μm. (**G**,**H**) Western blot analysis of YAP and GPX4 protein expression in A549 cells with YAP overexpression. **** *p* < 0.0001, *** *p* < 0.001, ** *p* < 0.01, * *p* < 0.05 vs. NC and ^###^ *p* < 0.001, ^##^ *p* < 0.01, ^#^ *p* < 0.05 vs. SAA (100 μM).

To conclusively demonstrate that YAP is the critical functional mediator of SAA, we constructed an A549 cell model with transient YAP overexpression (YAP-OE). The data showed that overexpression of YAP reduced the suppressive effect of SAA on A549 cell viability (Figure 6A). Transwell invasion assays provided further evidence that YAP overexpression partially reversed the inhibition of A549 cell invasion induced by SAA (Figure 6B,C). Separately, flow cytometry combined with Annexin V-FITC/PI staining showed that YAP overexpression led to a marked decrease in both early and late apoptotic cells after SAA treatment (Figure 6D,E). Next, we explored whether YAP is involved in SAA-induced ferroptosis. Notably, YAP overexpression effectively attenuated the SAA-induced accumulation of lipid peroxides (Figure 6F,I). Furthermore, Western blot analysis revealed that YAP overexpression partially counteracted the reduction in YAP and GPX4 expression induced by SAA (Figure 6G,H). Collectively, these findings confirm that YAP serves as a critical mediator for SAA to exert its antitumor and ferroptosis-promoting effects in A549 cells.

### 2.7. SAA Suppresses NSCLC Progression by Inducing Ferroptosis In Vivo

**Figure 7:**
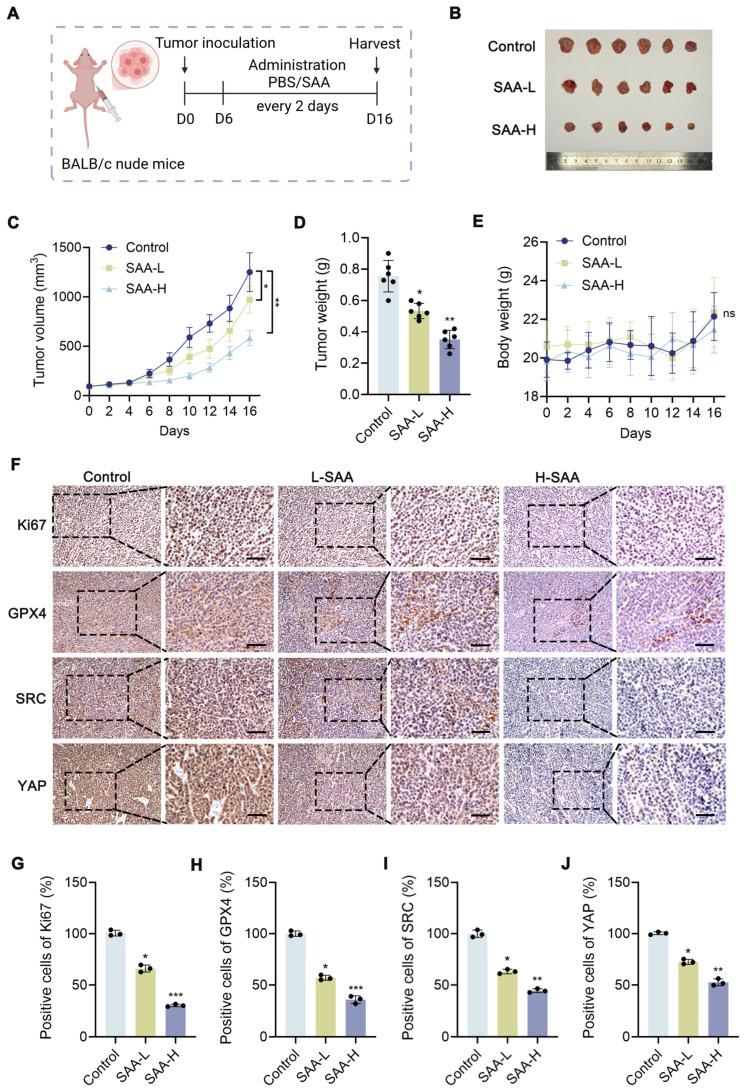
SAA suppresses the development of NSCLC by inducing ferroptosis. (**A**) BALB/c nude mice were administered PBS, low-dose SAA (50 mg/kg, SAA-L), or high-dose SAA (100 mg/kg, SAA-H) every 2 days. (**B**) Representative images of tumor tissues. (**C**) Quantification of tumor volume. (**D**) Assessment of tumor weight. (**E**) Throughout the dosing period, changes in body weight were recorded for the nude mice. (**F**–**J**) IHC analysis of Ki67, GPX4, SRC, and YAP expression. Scale bar: 50 μm. *** *p* < 0.001, ** *p* < 0.01, * *p* < 0.05 vs. control.

For in vivo assessment of SAA’s antitumor efficacy, nude mice were implanted with subcutaneous xenografts. Starting from day 6, the mice were randomized into three groups and administered PBS, low-dose SAA (50 mg/kg, SAA-L), or high-dose SAA (100 mg/kg, SAA-H) intraperitoneally every other day (Figure 7A). Significant tumor growth inhibition was detected in A549 xenografts treated with low and high doses of SAA, accompanied by reductions in tumor volume and weight (Figure 7B–D), while no significant differences in body weight were detected when comparing the control group with the groups treated with SAA (Figure 7E). Furthermore, IHC analysis revealed that the Ki67-positive cell rate decreased significantly following SAA intervention, indicating that SAA exerts a dose-dependent suppressive effect on in vivo tumor growth (Figure 7F,G). Concurrently, the expression of GPX4, SRC, and YAP was significantly reduced following SAA intervention (Figure 7F,H–J). Collectively, these findings demonstrate that SAA can suppress in vivo tumor growth by triggering cellular ferroptosis processes.

### 2.8. SAA Inhibits the Growth of Patient-Derived Lung Cancer Organoids

**Figure 8:**
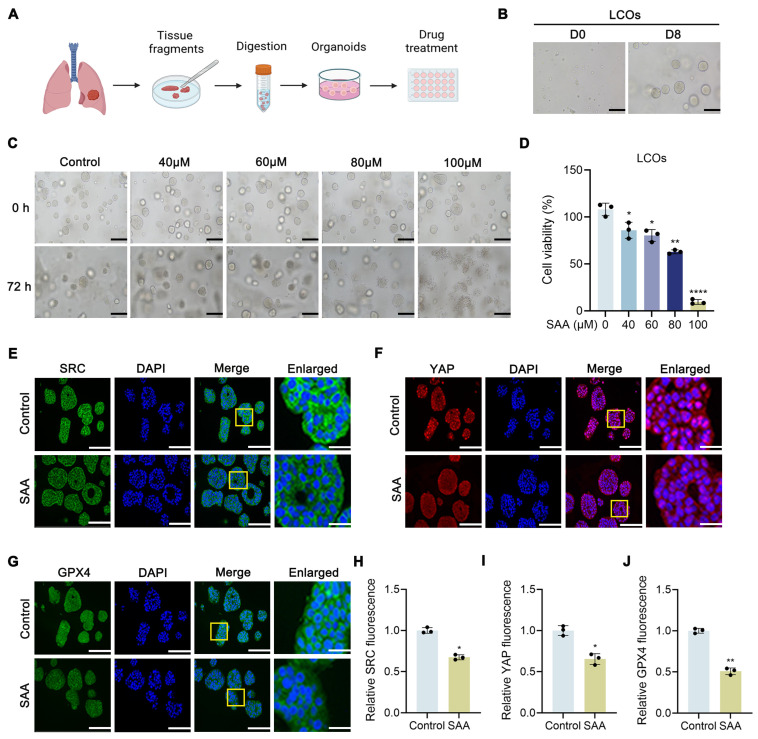
SAA inhibits the growth of patient-derived lung cancer organoids. (**A**,**B**) Diagram of patient-derived lung cancer organoid construction. (**C**) Representative photograph of SAA-treated lung cancer organoids. Scale bar: 100 μm. (**D**) CCK-8 assessed the viability of lung cancer organoids after SAA treatment. (**E**–**J**) IF staining of SRC, YAP and GPX4. Scale bar: 50 μm. **** *p* < 0.0001, ** *p* < 0.01, * *p* < 0.05 vs. control.

Lung cancer organoids (LCOs) exhibit histological features similar to those of the primary tumor tissue. We evaluated the therapeutic efficacy of SAA using patient-derived LCOs (Figure 8A,B). In patient-derived LCOs, SAA significantly inhibited organoid proliferation and disrupted their structural integrity (Figure 8C). CCK-8 assays further validated the inhibitory effect of SAA on LCO cell viability (Figure 8D). Additionally, immunofluorescence analysis revealed that SAA treatment decreased the expression of SRC and GPX4 in the cytomembrane and cytoplasm in LCOs and significantly decreased the nuclear translocation of YAP (Figure 8E–J). Taken together, these results indicate that SAA effectively inhibits LCO progression via the SRC/YAP/GPX4 axis.

## 3. Discussion

Our study identified SAA as a potent antitumor substance in NSCLC that acts by triggering ferroptosis through the inhibition of the SRC/YAP/GPX4 axis. Specifically, we demonstrated that SAA directly targets SRC and YAP, disrupting their interaction and inhibiting SRC-mediated activation and nuclear translocation of YAP. This suppression subsequently leads to the downregulation of GPX4, a master regulator of ferroptosis, resulting in lethal lipid peroxidation and iron accumulation. Through a combination of cellular and molecular experiments and animal xenograft models, this work presents a solid foundation for using SAA as a treatment to counteract NSCLC cell evasion of conventional apoptotic mechanisms.

Extracted from the roots of *Salvia miltiorrhiza Bunge*, the phenolic acid compound Salvianolic acid A (SAA) can suppress the growth of several cancer cell lines, such as HeLa, DU145, H1975, and A549. Previous research mainly attributed the antitumor effects of SAA to its regulation of MMP-2, the FAK/SRC/ERK signaling pathways, and the induction of PTEN-mediated apoptosis [21,27,28]. Despite these findings, the in vivo efficacy of SAA and its potential to engage ferroptosis remained largely unexplored. This work further enriches current research insights, confirms that SAA can effectively suppress the tumor growth of NSCLC in xenograft models, and reveals a novel mechanism centered on ferroptosis. It is particularly worth noting that SAA targets SRC autophosphorylation at Tyr416, thereby disrupting the SRC–YAP interaction, which differentiates its mechanism from the previously described apoptosis pathway.

Ferroptosis is a newly characterized form of regulated cell death, which is now regarded as a promising method for treating malignant tumors, including lung cancer [29,30,31]. The characteristics of this process include glutathione depletion, iron accumulation, and lipid peroxidation [32]. In addition, the persistence of drug-resistant cancer cells is heavily reliant on GPX4, a molecule that plays a central role in ferroptosis, rendering it a viable target for exploring and designing new antitumor treatment schemes [33,34]. However, evidence that SAA induces ferroptosis to suppress NSCLC cell growth remains scarce. Furthermore, the exact mechanism through which SAA suppresses NSCLC growth is poorly understood, underscoring the novelty and significance of investigating SAA-induced ferroptosis. In this study, we observed that inhibitors of ferroptosis scavengers could block cell death induced by SAA. More importantly, SAA effectively increased intracellular Fe^2+^ levels, ROS accumulation, and lipid peroxidation. Crucially, these alterations were mostly nullified by the ferroptosis-specific inhibitor Ferrostatin-1 (Fer-1), confirming that ferroptosis is the primary executioner of SAA-induced cell death in NSCLC. These findings provide a novel perspective for investigating SAA-induced ferroptosis in NSCLC.

SRC, a non-receptor tyrosine kinase, is extensively recognized for its involvement in the development and metastasis of various types of cancer [35]. It is worth noting that studies have shown that SRC family kinases can enhance the efficacy of osimertinib and inhibit drug resistance in patients with advanced EGFR-mutant NSCLC [36]. Moreover, studies have shown that inhibiting SRC can improve the antitumor efficacy of erlotinib-resistant NSCLC [37]. YAP is an essential downstream effector in the Hippo pathway. Studies have found that it can trigger various malignant behaviors in NSCLC, including tumor growth, metastasis, and therapy resistance [38,39,40]. Clinical analyses of NSCLC specimens frequently showed that the expression level of Hippo kinase LATS1 decreased, while the expression of YAP increased, which was associated with poor prognosis [41,42]. Furthermore, mouse model studies indicate that YAP effectively suppresses the metastasis of NSCLC [40,43,44]. There is sufficient evidence that SRC can activate YAP through direct phosphorylation or inhibition of Hippo kinase, thus promoting cell survival and inhibiting apoptosis [45,46,47,48]. Although previous reports have linked the SRC-YAP module to chemotherapy resistance and metastasis, our study reveals its pivotal role in regulating ferroptosis sensitivity. We observed that SAA blocked the nuclear translocation of YAP by inhibiting SRC-mediated phosphorylation, resulting in a significant decrease in GPX4 expression. This echoes the latest evidence in the context of other studies. Recent evidence has indicated that senescent endothelial cells may promote the progression of gastric cancer through suppression of ferroptosis mediated by the EGFR/SRC/YAP1/GPX4 signaling axis [26]. Interestingly, while small-molecule inhibitors such as dasatinib and verteporfin have been used to target SRC and YAP, respectively, SAA has a unique dual-targeting profile that effectively collapses this axis. The dependence of the SRC/YAP module on GPX4 underscores the complexity of ferroptosis control and suggests that the efficacy of SAA may be particularly effective in tumors with high baseline SRC or YAP activity.

In addition to conventional cell lines and animal models, we evaluated the efficacy of SAA in patient-derived LCOs, which replicate the histological structure, cellular heterogeneity and drug response characteristics of primary tumor tissue. In LCOs, SAA significantly inhibited organoid proliferation, disrupted structural integrity, decreased SRC and GPX4 expression, and blocked YAP nuclear translocation. These results provide compelling preclinical evidence to support the clinical translatability of SAA to the treatment of NSCLC.

Although this study generally supports the potential efficacy of SAA in the treatment of NSCLC, there are several limitations that should be considered. First, although we have preliminarily proved that the action of SAA in NSCLC involves its interaction with SRC and YAP, the exact binding site of SAA on SRC and YAP still needs to be further verified. Furthermore, future studies should employ CETSA, MST and SPR experiments to validate the combination of SAA with SRC and YAP. Finally, whether SAA affects other signaling pathways that trigger ferroptosis warrants additional investigation.

## 4. Materials and Methods

### 4.1. Cell Culture

Human lung cancer cell line A549 (ATCC, Manassas, VA, USA, CCL-185) and human normal lung epithelial cell line BEAS-2B (ATCC, CRL-3588) were grown in RPMI-1640 medium (VivaCell, Shanghai, China, C3010-0500) containing 10% FBS (Excell, Suzhou, China, FSP500) and 1% PSA (Solarbio, Beijing, China, P1410). A humidified 37 °C incubator containing 5% CO_2_ was used for all cultures.

### 4.2. Animal Experiments

All experimental protocols were approved by the Animal Ethics Committee of Chengdu University of Traditional Chinese Medicine (Approval No.: 2025806). BALB/c nude mice aged 6 to 8 weeks were obtained from Chengdu Yaokang Biotechnology Co., Ltd. (Chengdu, China). For tumor establishment, 5 × 10^6^ A549 cells were injected subcutaneously into the right scapula area of each mouse. Six days following cell inoculation, the animals were randomly assigned to three groups: control (PBS), low-dose SAA (50 mg/kg, SAA-L), and high-dose SAA (100 mg/kg, SAA-H). Thereafter, 200 μL of PBS or SAA was administered intraperitoneally every 2 days. During the whole treatment period, the tumor growth and body weight were evaluated every other day. The volume of each tumor was calculated with the following formula: (length × square of width)/2. At the end of the study, the mice were sacrificed, and the tumor specimens were promptly excised and photographed.

### 4.3. Organoid Culture

The surgically obtained tumor specimens were first rinsed, finely minced, and then digested at 37 °C for 1 h with 5 mg/mL collagenase type II (Gibco, Thermo Fisher Scientific, Waltham, MA, USA, 17101–015). Next, the disrupted tissue was filtered through a 70 μm cell strainer to obtain a single-cell suspension. Once this step is complete, the cells are resuspended in Matrigel (Corning, Corning, NY, USA, 356231) and seeded into 24-well culture plates. The gel was left to solidify at 37 °C for 15 min, and then complete organoid medium was supplemented to each well. During the culture period, the medium was changed every 3 days.

### 4.4. Reagents and Antibodies

Salvianolic acid A (CAS: 96574-01-5, purity: 98%) was purchased from Chengdu Lingliu Biotechnology (Chengdu, China). It was dissolved in DMSO to a concentration of 100 mM. Ferrostatin-1 (347174-05-4) and dasatinib (302962-49-8) were sourced from MCE (Monmouth Junction, NJ, USA). Z-VAD-FMK (C1201) was obtained from Beyotime Biotechnology (Shanghai, China). Verteporfin (S1786) was procured from Selleck Chemicals (Houston, TX, USA). Antibodies included anti-Ki67 (#9449), anti-GPX4 (67763-1-Ig), anti-p-SRC (Tyr416) (YM8471), anti-SRC (YM8399), anti-p-YAP1 (Ser127) (YM8552), and anti-YAP1 (66900-1-Ig).

### 4.5. Cell Viability Analysis

A549 cells were dispensed into 96-well culture plates and inoculated at 8 × 10^3^ cells per well. When the cell confluence approached 80%, they were exposed to varying concentrations of SAA for 24 h. To determine the cell survival rate, the CCK-8 assay was employed. In this experimental operation, each well received a mixture containing 10 μL of CCK-8 reagent and 90 μL of fresh RPMI-1640 medium. After 2 h of incubation at 37 °C, the plates were measured at 450 nm.

### 4.6. Wound Healing Assay

A549 cells were inoculated in 6-well culture plates, with each well receiving 5 × 10^5^ cells. The plates were incubated until cell coverage approached 90%. At that point, a sterile 10 μL pipette tip was employed to generate a uniform scratch across the confluent cell sheet. After gentle rinsing with PBS to remove dislodged cells, the cultures were exposed to varying concentrations of SAA. Photographs of the wound regions were subsequently obtained at 0, 24, and 48 h.

### 4.7. Transwell Assay

A total of 2 × 10^4^ A549 cells suspended in 200 µL of serum-free medium were added to the upper chamber, and 500 µL of complete medium was added to the lower chamber. After cells adhered, different concentrations of SAA were introduced into the upper compartment. Following a 48 h incubation period, the cells were fixed with 4% paraformaldehyde and subsequently stained with 0.1% crystal violet.

### 4.8. Flow Cytometry

Apoptosis was evaluated using the Annexin V-FITC/PI Apoptosis Assay Kit (Biosharp, Beijing, China, BL107B). In short, A549 cells were first seeded into a 6-well plate at a density of 5 × 10^5^ cells per well, and cultured until cell confluence reached approximately 80%. The cells were subsequently exposed to different concentrations of SAA and incubated for a period of 24 h. After that, they were harvested and rinsed twice with ice-cold PBS. After collection, the cells were stained in the dark for 15 min with Annexin V-FITC and propidium iodide (PI), and the apoptotic rate was finally analyzed using a flow cytometer (BD FACSCanto II, Franklin Lakes, NJ, USA).

### 4.9. Quantitative Real-Time PCR

Cellular total RNA was prepared with TRIzol reagent (Thermo Fisher Scientific, Waltham, MA, USA, 252612). The purified RNA samples were subsequently subjected to reverse transcription to generate cDNA with the Primescript First-Strand cDNA Synthesis SuperMix (Vazyme, Nanjing, China, R323-01). The qPCR assay was utilized to quantify mRNA levels using the SYBR Green Supermix (Bioground, Chongqing, China, BG0014). The specific primer sequences employed for this assay are presented in Table 1.

### 4.10. Transfection

The vector for YAP was obtained from GenePharma Biotechnology (Shanghai, China). Cell transfection with plasmids was carried out using Lipofectamine 3000 (Invitrogen, Thermo Fisher Scientific, Waltham, MA, USA, L3000015) following the manufacturer’s guidelines. Six hours post-transfection, the medium was replaced with fresh complete medium, and the cells were collected 48 h after the start of transfection.

### 4.11. Western Blot

Cell lysates were obtained with RIPA lysis buffer (Biosharp, BL504A), and protein content was measured using a BCA assay kit (Absin, Shanghai, China, abs9232). Equivalent amounts of protein from each sample were loaded and separated on 8–12% SDS-PAGE, followed by transfer onto PVDF membranes (Merck, Darmstadt, Germany, ISEQ00010). Following blocking, prepared membranes were incubated with corresponding primary antibodies. Target protein bands were finally visualized via a chemiluminescence imaging system (Tanon, Shanghai, China).

### 4.12. Co-Immunoprecipitation (Co-IP)

For the endogenous Co-IP assay, a total of 2 mg of protein lysate derived from cells was incubated with an anti-YAP antibody. Subsequently, the immune complexes were incubated with 40 μL of protein A/G agarose beads (Abmart, Shanghai, China, A10001). Following centrifugation, the beads were harvested and resuspended in the suitable IP lysate (Beyotime, Shanghai, China, P0013) and 5× loading buffer. Prior to Western blot analysis, the samples were denatured by heating at 100 °C for 10 min.

### 4.13. Analysis of Lipid Peroxidation

Intracellular lipid peroxidation was quantitatively assessed with a corresponding assay kit (Beyotime, S0043S). Briefly, A549 cells were incubated with BODIPY 581/591 C11, followed by staining of the cell nuclei with Hoechst 33342 (Servicebio, Wuhan, China, G1127). Fluorescence images were taken with a confocal high-content imaging analysis system (ImageXpress Micro Confocal, San Jose, CA, USA).

### 4.14. FerroOrange Staining

FerroOrange (Beyotime, S1070S) is a fluorescent probe for staining live cells and is used to measure intracellular Fe^2+^ ion content. A549 cells were incubated with FerroOrange, after which their cell nuclei were stained with Hoechst 33342. To observe intracellular Fe^2+^ levels, fluorescence images were captured using a high-content confocal imaging system.

### 4.15. ROS Measurement

The intracellular accumulation of ROS was assessed using DCFH-DA, a fluorescent indicator (Solarbio, CA1410). A549 cells were treated with DCFH-DA diluted in serum-free culture solution. The treated cells were later collected and examined through flow cytometry (BD FACSCanto II, USA) to measure fluorescence intensity.

### 4.16. Network Pharmacology Analysis

We obtained SAA-related targets from the Swiss Target Predictions (https://swisstargetprediction.ch/), Pharmmapper (https://www.lilab-ecust.cn/pharmmapper/, accessed on 11 December 2025), and TCMSP (https://www.tcmsp-e.com/) databases. We obtained the standard gene names and UniProt IDs of target proteins from UniProt (https://www.uniprot.org/), restricting the species to *Homo Sapiens*. Information on target genes associated with NSCLC was collected from the GeneCards (https://www.genecards.org/), DrugBank (https://go.drugbank.com/), and OMMI (https://www.omim.org/) databases. We merged the search results from these databases, removed duplicate entries, and obtained the final set of target genes for NSCLC. We then used Venny 2.1 to plot a Venn diagram of the SAA- and NSCLC-associated targets. The intersection of SAA and NSCLC-related targets was determined, and the intersection targets were input into the STRING database (https://cn.string-db.org/) to construct a PPI network. The minimum interaction score was set to 0.9, and disconnected targets were removed. Subsequently, the PPI network was visualized and analyzed using Cytoscape 3.10.9, with core genes screened based on the CytoNAC plugin. GO and KEGG analyses were performed using the Metascape database (https://metascape.org).

### 4.17. Molecular Docking

Molecular docking studies were carried out using AutoDock Vina version 1.5.7. The 3D structural models of SRC (PDB: 1Y57) and YAP (PDB: 9FZA) were retrieved from the RCSB Protein Data Bank, while the molecular structure of SAA was sourced from the PubChem database. Open Babel software 3.1.1 was employed to finish the conversion of file formats. Discovery Studio 2021 and PyMOL 2.2.0 were used to visualize the optimal protein–ligand complexes generated from these processes.

### 4.18. Immunofluorescence (IF) Staining

After pre-fixing the cells with 4% paraformaldehyde, perform permeabilization (0.1% Triton X-100) and blocking (5% goat serum). Next, incubate the cells with the selected primary antibodies, wash three times with PBS, and finally incubate with the fluorescently labeled secondary antibodies. Nuclear counterstaining was carried out with Hoechst 33342, and the fluorescence signals were subsequently visualized using a confocal high-content imaging analysis system.

### 4.19. Immunohistochemistry (IHC) Staining

Tumor specimens were initially preserved in 4% paraformaldehyde, embedded in paraffin and sectioned. After that, the sections were incubated with specific primary antibodies, followed by treatment with a biotinylated secondary antibody and finally with ABC solution. Following staining with DAB solution, the expression of the target protein was visualized and stained with hematoxylin.

### 4.20. Statistical Analysis

Data are expressed as means ± standard deviation (SD). Statistical analyses were performed using GraphPad Prism 10.0. Group differences were evaluated by Student’s *t*-test or one-way analysis of variance (ANOVA). Differences at *p* < 0.05 were considered statistically significant.

## 5. Conclusions

In conclusion, this study demonstrates that SAA induces ferroptosis through the SRC/YAP/GPX4 axis, thus inhibiting the progression of NSCLC. Mechanistically, SAA functions as an SRC kinase inhibitor by blocking SRC autophosphorylation at Tyr416, thereby disrupting the SRC-YAP interaction, inhibiting YAP nuclear translocation, and ultimately downregulating GPX4 expression to trigger ferroptotic cell death. These results offer a new understanding of the antitumor action of SAA and underscore its potential as a therapeutic strategy for NSCLC.

## Figures and Tables

**Table 1:** The following primer sequences were utilized for qRT-PCR.

Gene Name	Forward (3′-5′)	Reverse (3′-5′)
GPX4	ACAAGAACGGCTGCGTGGTGAA	GCCACACACTTGTGGAGCTAGA
SRC	TGGCAAGATCATCAGACGG	GGCACCTTTCGTGGTCTCAC
YAP	GAACTCGGCTTCAGGTCCTC	GGTTCATGGCAAAACGAGGG
GAPDH	AGAAGGCTGGGGCTCATTTG	AGGGGCCATCCACAGTCTTC

## Data Availability

The data that support the findings of this study are available from the corresponding author upon reasonable request.

## Supplementary Materials

The following supporting information can be downloaded at: https://www.mdpi.com/article/10.3390/ijms27146265/s1.

## Abbreviations

The following abbreviations are used in this manuscript:

NSCLC	Non-small-cell lung cancer
SAA	Salvianolic acid A
KEGG	Kyoto encyclopedia of genes and genomes
GO	Gene ontology
ROS	Reactive oxygen species
DCFH-DA	2:7-Dichlorodi-hydrofluorescein diacetate
CCK-8	Cell counting kit-8
FBS	fetal bovine serum
PSA	penicillin-streptomycin-amphotericin B
Fer-1	Ferrostatin-1
VP	Verteporfin
IF	Immunofluorescence
IHC	Immunohistochemistry
GPX4	Glutathione peroxidase 4
YAP	Yes-associated protein
SRC	SRC proto-oncogene, non-receptor tyrosine kinase

## References

[1] Zhang Y., Luo G., Etxeberria J., Hao Y. (2021). Global Patterns and Trends in Lung Cancer Incidence: A Population-Based Study. J. Thorac. Oncol..

[2] Wang M., Herbst R.S., Boshoff C. (2021). Toward Personalized Treatment Approaches for Non-Small-Cell Lung Cancer. Nat. Med..

[3] Bagchi S., Yuan R., Engleman E.G. (2021). Immune Checkpoint Inhibitors for the Treatment of Cancer: Clinical Impact and Mechanisms of Response and Resistance. Annu. Rev. Pathol..

[4] Garon E.B., Hellmann M.D., Rizvi N.A., Carcereny E., Leighl N.B., Ahn M.-J., Eder J.P., Balmanoukian A.S., Aggarwal C., Horn L. (2019). Five-Year Overall Survival for Patients with Advanced Non-Small-Cell Lung Cancer Treated with Pembrolizumab: Results from the Phase I KEYNOTE-001 Study. J. Clin. Oncol..

[5] Jackson C.M., Choi J., Lim M. (2019). Mechanisms of Immunotherapy Resistance: Lessons from Glioblastoma. Nat. Immunol..

[6] Dixon S.J., Lemberg K.M., Lamprecht M.R., Skouta R., Zaitsev E.M., Gleason C.E., Patel D.N., Bauer A.J., Cantley A.M., Yang W.S. (2012). Ferroptosis: An Iron-Dependent Form of Nonapoptotic Cell Death. Cell.

[7] Wu Y., Li H., Yue K., Jing C., Duan Y. (2025). Ferroptosis in Cancer: Metabolism, Mechanisms and Therapeutic Prospects. Mol. Cancer.

[8] Ye Z., Xie B., Tao Y., Xiao D. (2025). Mechanism of Ferroptosis and Its Role in Disease Development. Int. J. Biol. Sci..

[9] Wahida A., Conrad M. (2025). Decoding Ferroptosis for Cancer Therapy. Nat. Rev. Cancer.

[10] Toyokuni S., Kong Y., Maeda Y., Lyu Q., Ohara Y., Sato K., Motooka Y., Nakamura K., Tanaka H. (2025). Ferroptosis and Cancer: When Iron Turns against Tumors. Cell. Mol. Life Sci..

[11] Dos Santos A.F., Fazeli G., Xavier da Silva T.N., Friedmann Angeli J.P. (2023). Ferroptosis: Mechanisms and Implications for Cancer Development and Therapy Response. Trends Cell Biol..

[12] Rishi G., Huang G., Subramaniam V.N. (2021). Cancer: The Role of Iron and Ferroptosis. Int. J. Biochem. Cell Biol..

[13] Fang C.-Y., Wu C.-Z., Chen P.-N., Chang Y.-C., Chuang C.-Y., Lai C.-T., Yang S.-F., Tsai L.-L. (2018). Antimetastatic Potentials of Salvianolic Acid A on Oral Squamous Cell Carcinoma by Targeting MMP-2 and the c-Raf/MEK/ERK Pathway. Environ. Toxicol..

[14] Pei R., Si T., Lu Y., Zhou J.X., Jiang L. (2018). Salvianolic Acid A, a Novel PI3K/Akt Inhibitor, Induces Cell Apoptosis and Suppresses Tumor Growth in Acute Myeloid Leukemia. Leuk. Lymphoma.

[15] Zhang Q., Wang S., Yu Y., Sun S., Zhang Y., Zhang Y., Yang W., Li S., Qiao Y. (2016). Salvianolic Acid A, as a Novel ETA Receptor Antagonist, Shows Inhibitory Effects on Tumor In Vitro. Int. J. Mol. Sci..

[16] Yang Y., Zhang L., La X., Li Z., Li H., Guo S. (2019). Salvianolic Acid A Inhibits Tumor-Associated Angiogenesis by Blocking GRP78 Secretion. Naunyn-Schmiedeberg’s Arch. Pharmacol..

[17] Zhang T., Xu J., Li D., Chen J., Shen X., Xu F., Teng F., Deng Y., Ma H., Zhang L. (2014). Salvianolic Acid A, a Matrix Metalloproteinase-9 Inhibitor of Salvia Miltiorrhiza, Attenuates Aortic Aneurysm Formation in Apolipoprotein E-Deficient Mice. Phytomedicine.

[18] Kebebe D., Wu Y., Zhang B., Yang J., Liu Y., Li X., Ma Z., Lu P., Liu Z., Li J. (2019). Dimeric c(RGD) Peptide Conjugated Nanostructured Lipid Carriers for Efficient Delivery of Gambogic Acid to Breast Cancer. Int. J. Nanomed..

[19] Ye T., Chen R., Zhou Y., Zhang J., Zhang Z., Wei H., Xu Y., Wang Y., Zhang Y. (2022). Salvianolic Acid A (Sal A) Suppresses Malignant Progression of Glioma and Enhances Temozolomide (TMZ) Sensitivity via Repressing Transgelin-2 (TAGLN2) Mediated Phosphatidylinositol-3-Kinase (PI3K)/Protein Kinase B (Akt) Pathway. Bioengineered.

[20] Yang S., Xie H., Lin Q., Zhou L., Liu J., Fang Z., Tang Z., Yuan R., Su J., Li S. (2025). EM2, a Natural Product MST1/2 Kinase Activator, Suppresses Non-Small Cell Lung Cancer via Hippo Pathway Activation. Adv. Sci..

[21] Chuang C.-Y., Ho Y.-C., Lin C.-W., Yang W.-E., Yu Y.-L., Tsai M.-C., Yang S.-F., Su S.-C. (2020). Salvianolic Acid A Suppresses MMP-2 Expression and Restrains Cancer Cell Invasion through ERK Signaling in Human Nasopharyngeal Carcinoma. J. Ethnopharmacol..

[22] Song X., Zhou Z., Elmezayen A., Wu R., Yu C., Gao B., Minna J.D., Westover K.D., Zeh H.J., Kroemer G. (2024). SRC Kinase Drives Multidrug Resistance Induced by KRAS-G12C Inhibition. Sci. Adv..

[23] Tian H., Zhao D., Zhou Z., Kim A., Huang H., Lee Y.J., Qu Z., Kang R., Zeh H.J., Westover K.D. (2026). SRC at the Crossroads of KRAS Inhibitor Resistance: Mechanisms and Therapeutic Opportunities. Cancer Lett..

[24] Ke A., Yang W., Zhang W., Chen Y., Meng X., Liu J., Dai D. (2025). The Cardiac Glycoside Periplocymarin Sensitizes Gastric Cancer to Ferroptosis via the ATP1A1-Src-YAP/TAZ-TFRC Axis. Phytomedicine.

[25] Shen X.-J., Wei H.-L., Mo X.-C., Mo X.-X., Li L., He J.-C., Wei X.-Y., Qin X.-J., Xing S.-P., Luo Z. (2024). Adaptor Protein CEMIP Reduces the Chemosensitivity of Small Cell Lung Cancer via Activation of an SRC-YAP Oncogenic Module. Acta Pharmacol. Sin..

[26] Zhang Y., Ren Y., Wang Z., Zhang X., Li X., Yu Y., Qian L., Xiong Y. (2025). Exosomal SLC1A5 from Senescent Endothelial Cells Promotes Gastric Cancer Progression by Dampening Ferroptosis via the EGFR/SRC/YAP1/GPX4 Signaling. Free Radic. Biol. Med..

[27] Bi L., Chen J., Yuan X., Jiang Z., Chen W. (2013). Salvianolic Acid A Positively Regulates PTEN Protein Level and Inhibits Growth of A549 Lung Cancer Cells. Biomed. Rep..

[28] Tang X.-L., Yan L., Zhu L., Jiao D.-M., Chen J., Chen Q.-Y. (2017). Salvianolic Acid A Reverses Cisplatin Resistance in Lung Cancer A549 Cells by Targeting C-Met and Attenuating Akt/mTOR Pathway. J. Pharmacol. Sci..

[29] Lei G., Zhuang L., Gan B. (2022). Targeting Ferroptosis as a Vulnerability in Cancer. Nat. Rev. Cancer.

[30] Wang Z., Shen N., Wang Z., Yu L., Yang S., Wang Y., Liu Y., Han G., Zhang Q. (2024). TRIM3 Facilitates Ferroptosis in Non-Small Cell Lung Cancer through Promoting SLC7A11/xCT K11-Linked Ubiquitination and Degradation. Cell Death Differ..

[31] Zhou Q., Meng Y., Li D., Yao L., Le J., Liu Y., Sun Y., Zeng F., Chen X., Deng G. (2024). Ferroptosis in Cancer: From Molecular Mechanisms to Therapeutic Strategies. Signal Transduct. Target. Ther..

[32] Yang W.S., SriRamaratnam R., Welsch M.E., Shimada K., Skouta R., Viswanathan V.S., Cheah J.H., Clemons P.A., Shamji A.F., Clish C.B. (2014). Regulation of Ferroptotic Cancer Cell Death by GPX4. Cell.

[33] Hangauer M.J., Viswanathan V.S., Ryan M.J., Bole D., Eaton J.K., Matov A., Galeas J., Dhruv H.D., Berens M.E., Schreiber S.L. (2017). Drug-Tolerant Persister Cancer Cells Are Vulnerable to GPX4 Inhibition. Nature.

[34] Mikubo M., Inoue Y., Liu G., Tsao M.-S. (2021). Mechanism of Drug Tolerant Persister Cancer Cells: The Landscape and Clinical Implication for Therapy. J. Thorac. Oncol..

[35] Roskoski R. (2015). Src Protein-Tyrosine Kinase Structure, Mechanism, and Small Molecule Inhibitors. Pharmacol. Res..

[36] Ichihara E., Westover D., Meador C.B., Yan Y., Bauer J.A., Lu P., Ye F., Kulick A., de Stanchina E., McEwen R. (2017). SFK/FAK Signaling Attenuates Osimertinib Efficacy in Both Drug-Sensitive and Drug-Resistant Models of EGFR-Mutant Lung Cancer. Cancer Res..

[37] Yuan M., Xu L.-F., Zhang J., Kong S.-Y., Wu M., Lao Y.-Z., Zhou H., Zhang L., Xu H. (2019). SRC and MEK Co-Inhibition Synergistically Enhances the Anti-Tumor Effect in Both Non-Small-Cell Lung Cancer (NSCLC) and Erlotinib-Resistant NSCLC. Front. Oncol..

[38] Bu F., Zhang Y., Zhao N., Tian X., Xu Y. (2023). Ezrin Regulates the Progression of NSCLC by YAP and PD-L1. Clin. Transl. Oncol..

[39] Sharma R., Sharma S., Shriwas P., Mehta L., Vu A.H., Mouw J.K., Koo J., Huang C., Matsuk V.Y., Tucker-Burden C. (2024). Intra-Tumoral YAP and TAZ Heterogeneity Drives Collective NSCLC Invasion That Is Targeted by SUMOylation Inhibitor TAK-981. iScience.

[40] Wang Y., Dong Q., Zhang Q., Li Z., Wang E., Qiu X. (2010). Overexpression of Yes-Associated Protein Contributes to Progression and Poor Prognosis of Non-Small-Cell Lung Cancer. Cancer Sci..

[41] Ye X.-Y., Luo Q.-Q., Xu Y.-H., Tang N.-W., Niu X.-M., Li Z.-M., Shen S.-P., Lu S., Chen Z.-W. (2015). 17-AAG Suppresses Growth and Invasion of Lung Adenocarcinoma Cells via Regulation of the LATS1/YAP Pathway. J. Cell. Mol. Med..

[42] Dubois F., Keller M., Calvayrac O., Soncin F., Hoa L., Hergovich A., Parrini M.-C., Mazières J., Vaisse-Lesteven M., Camonis J. (2016). RASSF1A Suppresses the Invasion and Metastatic Potential of Human Non-Small Cell Lung Cancer Cells by Inhibiting YAP Activation through the GEF-H1/RhoB Pathway. Cancer Res..

[43] Hsu P.-C., Tian B., Yang Y.-L., Wang Y.-C., Liu S., Urisman A., Yang C.-T., Xu Z., Jablons D.M., You L. (2019). Cucurbitacin E Inhibits the Yes-associated Protein Signaling Pathway and Suppresses Brain Metastasis of Human Non-small Cell Lung Cancer in a Murine Model. Oncol. Rep..

[44] Hsu P.-C., Miao J., Huang Z., Yang Y.-L., Xu Z., You J., Dai Y., Yeh C.-C., Chan G., Liu S. (2018). Inhibition of Yes-Associated Protein Suppresses Brain Metastasis of Human Lung Adenocarcinoma in a Murine Model. J. Cell. Mol. Med..

[45] Cui X., Morales R.-T.T., Qian W., Wang H., Gagner J.-P., Dolgalev I., Placantonakis D., Zagzag D., Cimmino L., Snuderl M. (2018). Hacking Macrophage-Associated Immunosuppression for Regulating Glioblastoma Angiogenesis. Biomaterials.

[46] He C., Mao D., Hua G., Lv X., Chen X., Angeletti P.C., Dong J., Remmenga S.W., Rodabaugh K.J., Zhou J. (2015). The Hippo/YAP Pathway Interacts with EGFR Signaling and HPV Oncoproteins to Regulate Cervical Cancer Progression. EMBO Mol. Med..

[47] Si Y., Ji X., Cao X., Dai X., Xu L., Zhao H., Guo X., Yan H., Zhang H., Zhu C. (2017). Src Inhibits the Hippo Tumor Suppressor Pathway through Tyrosine Phosphorylation of Lats1. Cancer Res..

[48] Yun M.R., Choi H.M., Lee Y.W., Joo H.S., Park C.W., Choi J.W., Kim D.H., Kang H.N., Pyo K.-H., Shin E.J. (2019). Targeting YAP to Overcome Acquired Resistance to ALK Inhibitors in ALK-Rearranged Lung Cancer. EMBO Mol. Med..

