# Supplementary material for: Salvianolic Acid A Induces Ferroptosis in Non-Small Cell Lung Cancer via the SRC/YAP/GPX4 Axis
Source: Int J Mol Sci. 2026 Jul 14;27(14):6265. doi: 10.3390/ijms27146265 (PMC13410247; doi:10.3390/ijms27146265)
Supplement: Supplementary file 1 [file ijms-27-06265-s001.zip › ijms-4409761-supplementary.pdf]

Supplementary Material

Salvianolic acid A induces ferroptosis in non-small cell lung cancer via the SRC/YAP/GPX4 axis

Ruyu Jiang [1,†], Haoshu Liu [1,†], Hairong Xiang [1], Xiaomeng Tang [2], Linfeng Zhao [1], Dawei Zeng [1], Yue Zhang [1], Jiazhen Xie [1], Yanju Gong [1,*] and Lan Yang [1,*]

Table S1. IC50 and Selectivity Index (SI) values of SAA in A549 and BEAS-2B cells.

Cell line	IC50 (μM)	SI
A549	58.85 (56.54-61.12)	2.86 (2.67-3.09)
BEAS-2B	168.6 (163.1-174.7)	–

Table S2. Molecular docking parameters for SRC and YAP.

Protein	Crystal code	Coordinates (x, y, z)	Size (x, y, z)
YAP	9FZA	(-10.829, -0.221, 43.906)	(72.45, 72.45, 72.45)
SRC	1Y57	(-3.456, 38.27, 38.334)	(74.55, 74.55, 74.55)

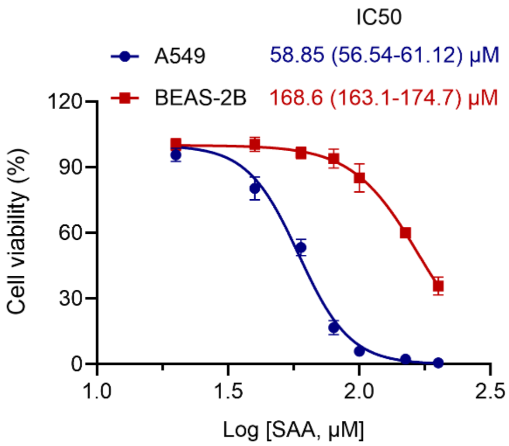

Figure S1. IC50 values of SAA in A549 and BEAS-2B cells.

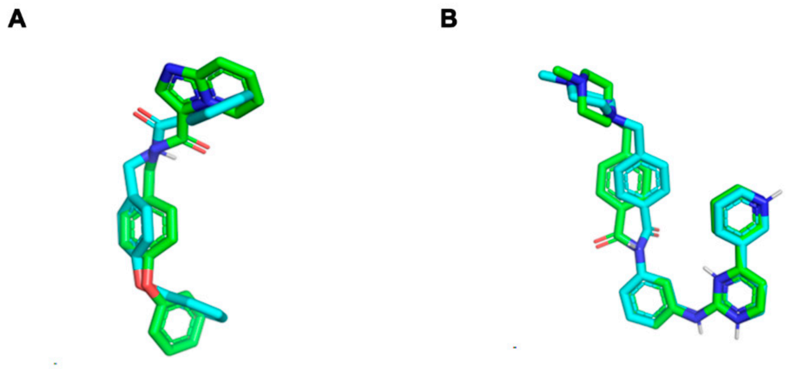

Figure S2. Validation of the docking by redocking of co-crystallized ligands. (A) Redocking validation of YAP with an RMSD of 1.987 Å. (B) Redocking validation of SRC with an RMSD of 0.644 Å.

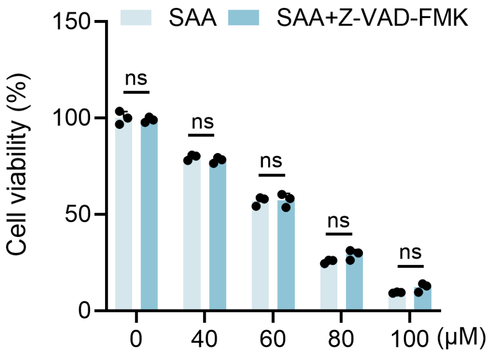

Figure S3. Treatment of A549 cells with SAA (0, 40, 60, 80 and 100 μM) with or without Z-VAD (20 μM) for 24 h, and subsequent cell viability detection.
